# Progress in the application of combined detection of urinary tumor DNA and exosomes in the classification, staging, and clinical management of bladder cancer: a review

**DOI:** 10.3389/fonc.2026.1745178

**Published:** 2026-05-12

**Authors:** Mengyun Wang, Jianchuan Chen, Cheng Wang, Yue He

**Affiliations:** 1 North Sichuan Medical College, Nanchong, China; 2 Department of Urology, Suining Central Hospital, Suining, China

**Keywords:** bladder cancer, exosomes, liquid biopsy, minimal residual disease, molecular classification, non-invasive diagnosis, precision medicine, urinary tumor DNA

## Abstract

Bladder cancer (BC) remains a major clinical challenge due to its high recurrence rate and biological heterogeneity. Traditional diagnostic methods, such as cystoscopy and urine cytology, are limited by invasiveness and low sensitivity for early or low-grade tumors. Recent advances in liquid biopsy have highlighted the potential of urinary tumor DNA (utDNA) and exosomes as non-invasive biomarkers for diagnosis, molecular classification, staging, and therapeutic monitoring. utDNA reflects the genomic and epigenetic alterations of BC with high concordance to tumor tissue, while urinary exosomes serve as carriers of proteins, RNA, and DNA that mirror tumor biology and microenvironmental interactions. The combined detection of utDNA and exosomes significantly enhances diagnostic sensitivity and specificity, facilitates early disease detection, and enables a dynamic monitoring of treatment response and recurrence. Standardization of sample collection, analytical platforms, and data interpretation, together with multicenter clinical validation, is crucial for clinical translation. Future research integrating multi-omics profiling and artificial intelligence is expected to establish urinary liquid biopsy as a key tool for precision medicine in BC management.

## 1 Clinical characteristics, diagnosis, and treatment status of BC classification and staging

### 1.1 Epidemiology and pathological classification of BC

Bladder cancer (BC) is one of the most common malignancies of the urinary system worldwide and is characterized by high incidence and recurrence rates, posing substantial challenges to public health and clinical management. A comprehensive review published in 2023 reported that BC ranks among the 10 most prevalent cancers globally, and its incidence continues to increase due to population growth, population aging, and major risk factors such as cigarette smoking (1). The incidence of BC is significantly higher in men than in women, and sex-related differences in disease prognosis have also been reported. Histologically, urothelial carcinoma (UC) represents the predominant pathological subtype of BC.

However, BC exhibits substantial histological heterogeneity and includes more than 40 recognized variants. These variants include squamous cell carcinoma, small cell carcinoma, sarcomatoid UC, micropapillary carcinoma, plasmacytoid carcinoma, urachal carcinoma, and adenocarcinoma (2). Many of these variants demonstrate more aggressive biological behavior and poorer prognosis than conventional UC, often requiring distinct therapeutic strategies. Therefore, accurate pathological classification is essential to guide clinical management. From a molecular pathology perspective, BC is broadly classified into non-muscle-invasive bladder cancer (NMIBC) and muscle-invasive bladder cancer (MIBC). NMIBC accounts for approximately 75% of newly diagnosed cases and is characterized by a high recurrence rate of nearly 70%, with approximately 15% of patients progressing to muscle-invasive disease (3).

In contrast, MIBC demonstrates greater biological aggressiveness, higher metastatic potential, and significantly poorer clinical outcomes. At the molecular level, these two disease categories exhibit distinct oncogenic mechanisms. NMIBC is commonly associated with the activation of the RAS–MAPK signaling pathway and frequent mutations in FGFR3, RAS, and PI3K genes, typically involving relatively limited genomic alterations. In contrast, MIBC is characterized by extensive genomic instability and frequent alterations in the TP53 and RB tumor suppressor pathways (4).

Recent molecular classification studies have further identified six major molecular subtypes of BC, including neural-like, HER2-like, papillary-like, luminal-like, mesenchymal-like, and squamous-like tumors. These subtypes exhibit distinct biological characteristics, clinical outcomes, and therapeutic responses (5)—for example, the papillary-like subtype is enriched for FGFR3 mutations and is generally associated with favorable prognosis, whereas the squamous-like subtype is often characterized by the activation of immunosuppressive signaling pathways and poorer clinical outcomes. Ongoing studies on molecular biomarkers have revealed frequent genetic alterations in genes such as TERT, FGFR3, TP53, PIK3CA, and STAG2 as well as mutations affecting chromatin-remodeling pathways (6). In addition to genomic alterations, other molecular features—including urinary metabolites, proteins, and DNA methylation markers—have emerged as promising non-invasive biomarkers for the early diagnosis and prognostic assessment of BC (7, 8).

### 1.2 Staging standards, clinical diagnosis, and treatment workflow

The clinical staging of BC is primarily based on the tumor–node–metastasis (TNM) classification system established by the Union for International Cancer Control (UICC), in combination with the World Health Organization (WHO) tumor grading system. The TNM classification stratifies diseases according to the depth of tumor invasion (T), the presence of regional lymph node involvement (N), and distant metastasis (M). The 1997 TNM classification and the 1998 WHO grading system remain widely used in clinical practice (9).

Within this framework, NMIBC includes stages Ta (non-invasive papillary carcinoma), Tis (carcinoma *in situ*), and T1 (invasion of the lamina propria). MIBC refers to stage T2 or higher, in which tumor cells invade the muscular layer of the bladder wall or extend beyond it. An initial clinical evaluation of suspected BC commonly relies on patient symptoms, particularly gross hematuria, together with urinary cytology and imaging examinations such as ultrasonography and computed tomography (CT) urography (10). However, cystoscopy combined with transurethral resection of bladder tumor (TURBT) remains the gold standard for definitive diagnosis and pathological staging.

Treatment strategies are largely determined by tumor stage and grade. Patients with NMIBC are typically treated with TURBT, followed by intravesical therapy, including chemotherapy or immunotherapy such as Bacillus Calmette–Guérin (BCG), to reduce recurrence and progression risks (6). In contrast, the standard curative treatment for MIBC is radical cystectomy, often combined with neoadjuvant chemotherapy, which has been shown to improve overall survival (11). Recent advances in immunotherapy have significantly expanded the treatment options for BC. Immune checkpoint inhibitors have demonstrated substantial efficacy, particularly in patients with advanced or metastatic disease (10).

### 1.3 Limitations of traditional detection and management methods

Despite major advances in treatment, the diagnosis and surveillance of BC still rely heavily on cystoscopy and urinary cytology. Although cystoscopy remains the diagnostic gold standard, it is invasive, costly, and associated with patient discomfort.

Furthermore, its sensitivity for detecting small or low-grade tumors remains limited (12). Urinary cytology demonstrates high sensitivity for high-grade tumors, but its performance in detecting low-grade lesions is poor.

In addition, the interpretation of cytology results is highly dependent on operator expertise and is subject to interobserver variability (13). Conventional imaging modalities, including CT and magnetic resonance imaging (MRI), also have limitations. These techniques are relatively insensitive for detecting early lesions and often fail to accurately evaluate minimal invasion or residual disease after treatment (14, 15). To overcome these limitations, considerable research has focused on the development of non-invasive molecular biomarkers detectable in urine or blood. Several urinary biomarker assays—including nuclear matrix protein 22 (NMP22), bladder tumor antigen (BTA), and UroVysion fluorescence *in situ* hybridization (FISH)—have been approved by the US Food and Drug Administration (FDA).

However, their diagnostic performance remains insufficient to replace cystoscopy in routine clinical practice (16). Emerging studies have explored additional biomarkers, including urinary proteins, metabolites, and DNA methylation markers, which have demonstrated encouraging diagnostic potential. Nevertheless, most of these biomarkers still require large-scale prospective validation before clinical implementation (17).

### 1.4 Conceptual framework of urinary liquid biopsy in BC

Recent advances in liquid biopsy technologies have introduced new opportunities for non-invasive molecular profiling of BC. Among these approaches, the analysis of utDNA and urinary exosomes has received increasing attention. utDNA refers to tumor-derived DNA fragments that are released into urine through cellular apoptosis, necrosis, or active secretion. Because urine directly contacts bladder tumor lesions, utDNA often contains higher mutant allele fractions than plasma circulating tumor DNA (ctDNA), potentially enabling more sensitive detection of tumor-specific genetic alterations.

In parallel, urinary exosomes—small extracellular vesicles secreted by tumor and stromal cells—carry diverse molecular cargos, including DNA, RNA, proteins, and lipids. These vesicles protect their molecular contents from degradation and may reflect tumor–microenvironment interactions as well as intra-tumoral heterogeneity.

Importantly, utDNA and exosomes provide complementary molecular information. utDNA primarily reflects tumor genomic alterations, whereas exosomes capture broader biological signals related to tumor activity and microenvironmental interactions. Integrating genomic alterations detected by utDNA with extracellular-vesicle-derived molecular signatures may therefore overcome the limitations of single biomarker strategies. Consequently, combined detection approaches represent a biologically rational and potentially synergistic strategy for the comprehensive molecular profiling of BC.

### 1.5 Literature search strategy and review methodology

This review was conducted as a narrative review with a structured literature screening strategy. A comprehensive literature search was performed using PubMed, Web of Science, and Embase databases for studies published up to 2025. The primary search terms included combinations of “bladder cancer,” “urinary tumor DNA,” “utDNA,” “circulating tumor DNA,” “exosomes,” “extracellular vesicles,” “liquid biopsy,” “molecular classification,” “minimal residual disease,” and “recurrence monitoring.”

Studies were included if they met the following criteria: (1) investigation of utDNA and/or urinary exosomes in BC, (2) reporting of outcomes related to diagnosis, staging, molecular classification, prognosis, or disease monitoring, and (3) provision of original clinical or translational data. Reviews, case reports, purely technical studies without clinical correlation, and studies focusing exclusively on blood-based biomarkers were excluded, unless they were directly relevant for methodological comparison.

Particular emphasis was placed on studies evaluating the combined detection of utDNA and exosomes, as emerging evidence suggests that integration of genomic and extracellular-vesicle-derived biomarkers may improve diagnostic accuracy and better reflect tumor heterogeneity. Due to the heterogeneity of study designs and outcome measures, a formal meta-analysis was not performed. The findings were qualitatively synthesized instead, with attention to study design, cohort characteristics, and translational relevance.

## 2 Biological basis and advances in detection technologies of utDNA and exosomes

### 2.1 Sources and molecular characteristics of utDNA

utDNA refers to DNA fragments released from tumor cells within the urinary tract and subsequently detected in urine. These DNA fragments originate primarily from tumor cell apoptosis, necrosis, and active secretion processes. Because urine is in direct contact with bladder tumors, utDNA provides a highly tumor-enriched source of genetic material for molecular analysis. Compared with ctDNA detected in plasma, utDNA often demonstrates higher analytical sensitivity for urological malignancies. Numerous studies have shown that utDNA reflects the genomic landscape of BC, including a wide spectrum of molecular alterations such as point mutations, copy number variations (CNVs), gene fusions, and DNA methylation changes. Importantly, the molecular profile of utDNA shows high concordance with tumor tissue DNA, supporting its potential utility for non-invasive tumor profiling (18, 19).

Earlier studies primarily focused on detecting tumor DNA in urine through analyses of microsatellite instability, gene mutations, and promoter methylation. These investigations demonstrated significantly higher detection rates in patients with BC compared with healthy individuals, with specificity approaching 100%. These earlier findings established the theoretical basis for using utDNA in the early diagnosis and recurrence monitoring of BC (18, 20). With the development of high-throughput sequencing technologies, next-generation sequencing (NGS) has substantially expanded the analytical capability of utDNA detection. NGS-based approaches enable the comprehensive genomic profiling of utDNA and allow the identification of a broader spectrum of tumor-specific alterations—for example, Zhang et al. applied NGS to analyze utDNA from patients with BC and observed a strong concordance between utDNA and tumor tissue DNA in mutation frequency, tumor mutational burden (TMB), and tumor cell fraction. The assay achieved a sensitivity level of 86.7% and a specificity level of 99.3%, outperforming plasma ctDNA in diagnostic accuracy (19).

In addition to genomic alterations, the epigenetic features of utDNA have also been extensively investigated. Chen et al. developed the utMeMA methylation assay, which integrates multiple methylation markers for the early diagnosis and recurrence monitoring of BC. This assay achieved a sensitivity level of 90.0% and a specificity level of 83.1%. Notably, utMeMA demonstrated superior performance compared with conventional urine cytology and FISH in detecting early-stage tumors and minimal residual disease (MRD) (21). Beyond its diagnostic value, utDNA also provides insights into tumor heterogeneity. Because utDNA originates from tumor cells throughout the urinary tract and surrounding microenvironment, it may capture a more representative overview of tumor genomic diversity. Smith et al. compared urinary and plasma ctDNA in patients with renal tumors and found that urinary ctDNA more accurately reflected spatial tumor heterogeneity, highlighting the unique advantages of urine-based liquid biopsy for urological cancers (20).

**Figure 1:**
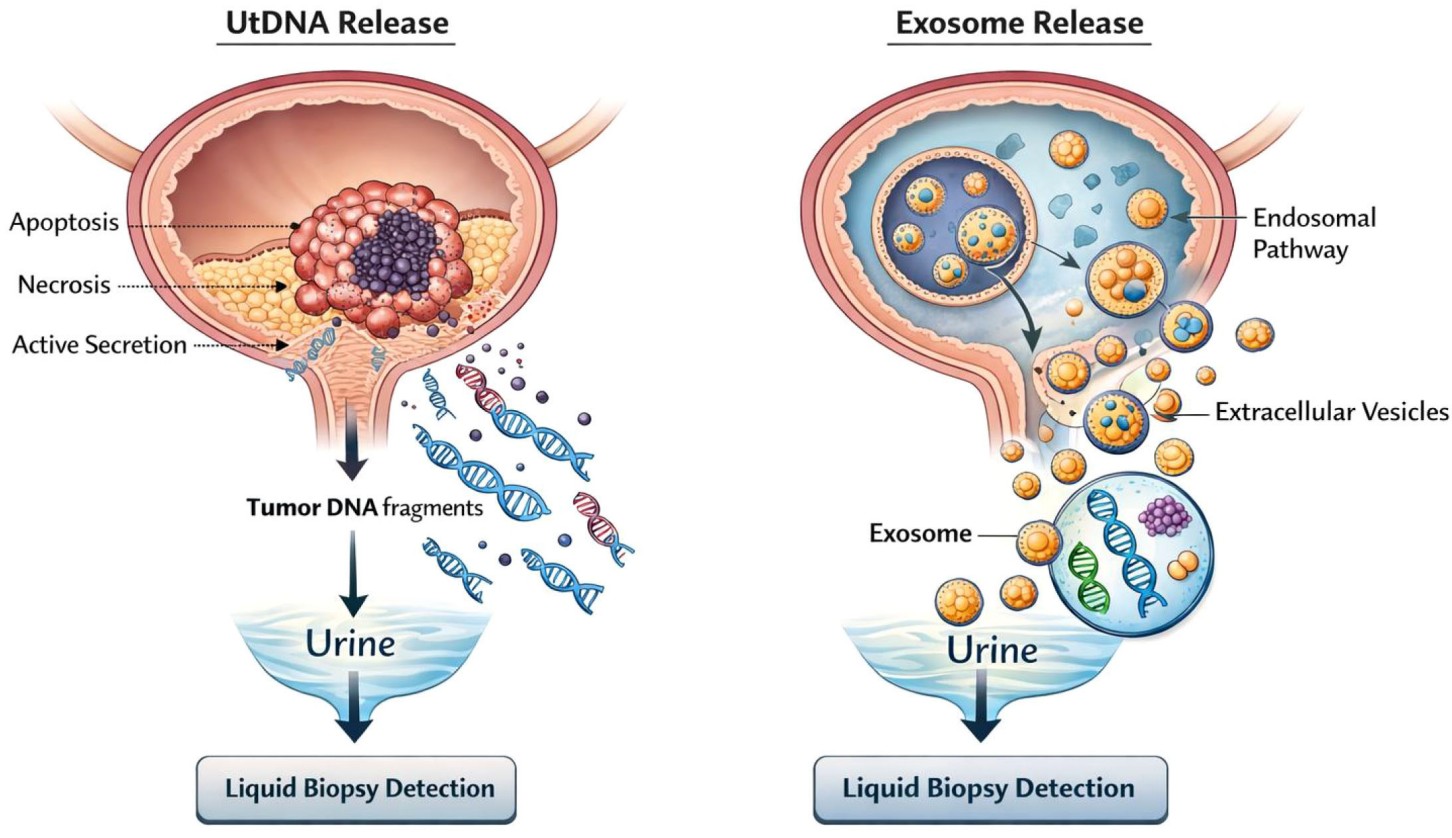
Biological origin of urinary tumor DNA (utDNA) and tumor-derived exosomes in bladder cancer.

Furthermore, dynamic changes in utDNA levels can be used to monitor therapeutic response and disease progression. Birkenkamp-Demtröder et al. reported that increases in ctDNA levels could precede radiological evidence of tumor recurrence, suggesting that ctDNA-based monitoring may provide an early indicator of disease relapse during postoperative surveillance (22). Collectively, these studies indicate that utDNA represents a promising non-invasive molecular biomarker for BC. By capturing both genetic and epigenetic alterations, utDNA provides valuable molecular information for early diagnosis, molecular classification, treatment monitoring, and recurrence surveillance. The biological origins of utDNA and urinary exosomes in BC are illustrated in **Figure 1**.

### 2.2 Biogenesis, isolation, and biomarkers of urinary exosomes

Exosomes are nanoscale extracellular vesicles with diameters ranging from approximately 30 to 150 nm. They are generated through the endosomal pathway and released into the extracellular environment when multivesicular bodies (MVBs) fuse with the plasma membrane. In urine, exosomes are primarily derived from epithelial cells lining the urinary tract as well as tumor cells. Urinary exosomes contain a wide variety of bioactive molecules, including proteins, messenger RNA (mRNA), microRNA (miRNA), long non-coding RNA (lncRNA), and DNA. Because these vesicles protect their molecular contents from enzymatic degradation, they provide a stable source of biomarkers for non-invasive cancer detection (23, 24). The biogenesis of exosomes begins with the inward budding of the endosomal membrane, forming early endosomes that subsequently mature into MVBs. These structures contain numerous intraluminal vesicles enriched with diverse biomolecules. When MVBs fuse with the plasma membrane, these vesicles are released into the extracellular environment as exosomes. Exosomes derived from BC cells play important roles in intercellular communication within the tumor microenvironment. They can mediate immune modulation, influence tumor cell proliferation, and promote tumor invasion and metastasis—for example, Beckham et al. demonstrated that exosomes derived from high-grade BC cells and from patients’ urine were enriched in the protein EDIL-3. This protein promotes tumor cell migration and angiogenesis through the activation of the epidermal growth factor receptor (EGFR) signaling pathway, suggesting potential diagnostic and biological significance for exosome-associated proteins (25). Several techniques have been developed to isolate urinary exosomes, including ultracentrifugation, density-gradient centrifugation, polymer-based precipitation, immunoaffinity capture, and microfluidic chip technologies. These methods differ in terms of purity, yield, processing time, and operational complexity.

Recent advances in proteomics and high-throughput mass spectrometry have enabled the comprehensive molecular profiling of urinary exosomes. These studies have identified multiple proteins differentially expressed in BC patients, providing a foundation for the development of multi-marker diagnostic models—for instance, Suh et al. used mass spectrometry to identify urinary exosomal proteins and constructed two diagnostic models that achieved AUROC values of 0.845 and 0.842, both outperforming traditional urine cytology (26).

In addition to protein biomarkers, nucleic acid components within urinary exosomes have also attracted increasing attention. Zhou et al. performed whole-exome sequencing and identified BC-related driver mutations within urinary exosomal DNA (exoDNA). Interestingly, some of these mutations were unique to urine-derived exosomes, suggesting that exoDNA may capture additional genomic features related to tumor heterogeneity (27).

Similarly, Murakami et al. used RNA sequencing to identify urinary exosomal mRNA biomarkers, including SLC2A1, GPRC5A, and KRT17, which were significantly upregulated in BC patients and demonstrated promising diagnostic performance (28). Because of their high stability and non-invasive accessibility, urinary exosomes are also attractive candidates to monitor disease progression. Eldh et al. reported that exosomes isolated from urine and bladder tissue retained cancer-associated proteomic signatures even in patients with pathological remission, suggesting potential roles in tumor recurrence and metastasis (29). Furthermore, Hiltbrunner et al. demonstrated that urinary exosome proteomic profiles could distinguish tumor-bearing from non-tumor regions of the bladder, indicating their involvement in remodeling the tumor microenvironment (30). Taken together, urinary exosomes serve as stable carriers of multiple bioactive molecules and represent an important source of non-invasive biomarkers for BC. The analysis of their molecular contents provides valuable insights for early detection, molecular characterization, and therapeutic monitoring.

### 2.3 Combined detection platforms and emerging analytical technologies

**Figure 2:**
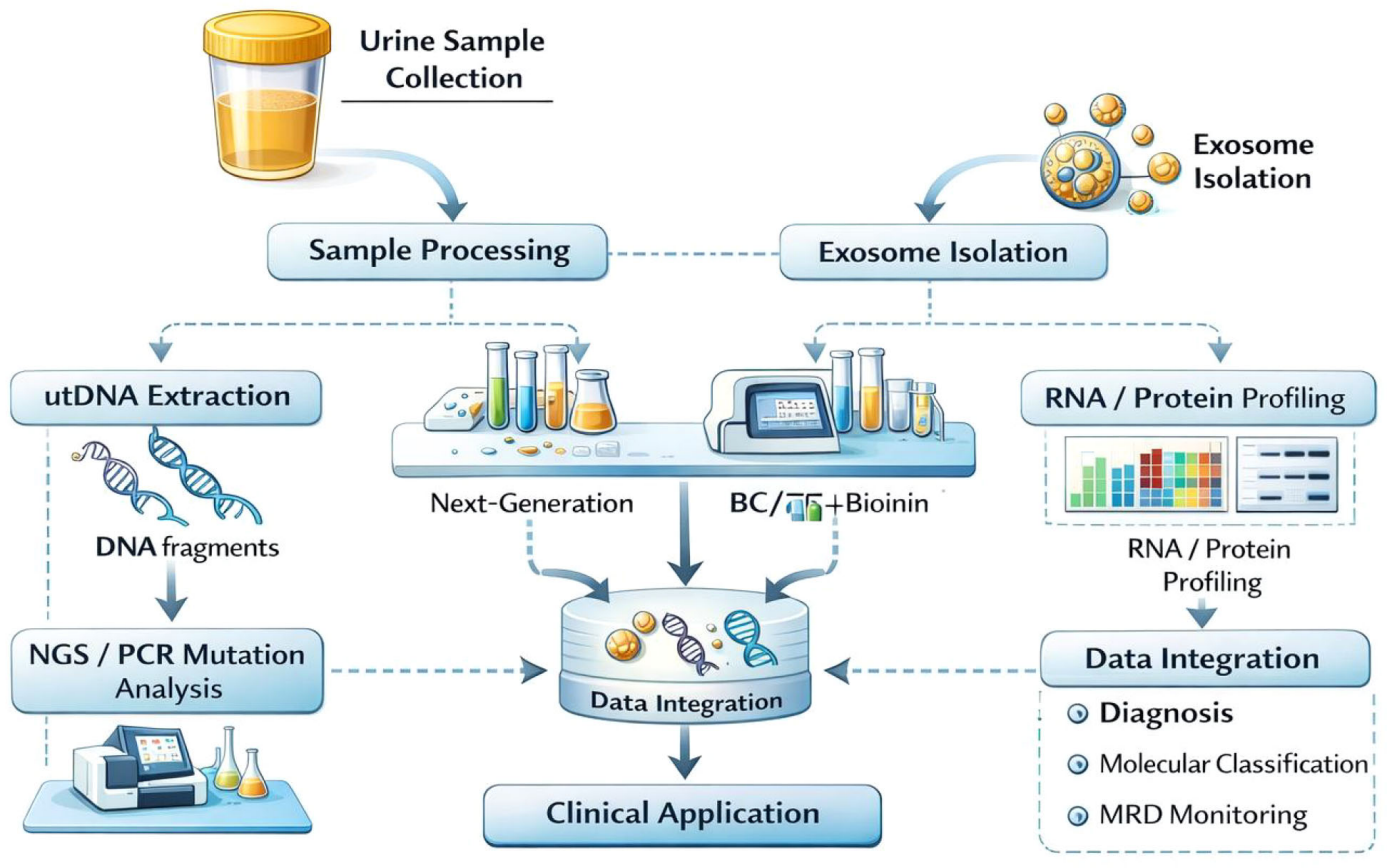
Workflow of combined utDNA and exosome detection for bladder cancer liquid biopsy.

A schematic workflow of the combined utDNA and exosome detection platform is presented in **Figure 2**. With advances in molecular diagnostic technologies, integrated detection strategies combining utDNA and exosome analysis have emerged as promising tools for the precision management of BC. These approaches integrate genomic information obtained from utDNA with protein and nucleic acid biomarkers derived from urinary exosomes, thereby improving analytical sensitivity and diagnostic performance. Dudley et al. developed a high-throughput sequencing method known as utDNA CAPP-Seq (uCAPP-Seq). This method uses the targeted capture of multiple tumor-specific mutations in utDNA and enables a highly sensitive detection of early-stage BC. In their study, a median of six tumor mutations was detected per sample, and the method significantly outperformed conventional diagnostic approaches (31). Chauhan et al. further expanded this strategy by combining ultra-low-pass whole-genome sequencing (ULP-WGS) with deep targeted sequencing (uCAPP-Seq). Using these approaches, the authors performed a multi-omics analysis of urinary cell-free DNA (cfDNA) and constructed a random forest prediction model for MRD. This model achieved a sensitivity level of 87% and successfully predicted progression-free survival and overall survival (32).

Similarly, Yang et al. developed the utLIFE-UC model, which integrates BC-specific gene mutations and extensive CNVs. This model achieved a sensitivity level of 92.8% and a specificity level of 96.0% for detecting UC, including both BC and upper urinary tract cancers.

Notably, its diagnostic performance exceeded that of conventional urine cytology and FISH for MRD monitoring (33). Other targeted detection platforms have also been reported. Kravchuk et al. reviewed the Uromonitor system, which detects hotspot mutations in TERT, FGFR3, and KRAS. The platform demonstrated a sensitivity level of 80.2% and a specificity level of 96.9%, markedly exceeding those of traditional urinary cytology (34). Parallel with sequencing-based technologies, significant progress has been made in analytical platforms and data processing approaches. Chen et al. summarized recent developments in urinary liquid biopsy technologies, highlighting the integration of microfluidic systems and artificial intelligence algorithms to improve sample processing, biomarker detection, and clinical interpretation (35). Lee et al. introduced the uAL100 assay, which employs targeted deep sequencing of urinary DNA to detect tumor-associated mutations. This approach achieved a sensitivity level of 83.7% and a specificity level of 100%, significantly reducing the need for unnecessary cystoscopy examinations (36).

In addition to genomic assays, exosome-based biomarker platforms have also been developed—for example, Suh et al. constructed a multi-protein urinary exosome detection model using high-throughput mass spectrometry, achieving an AUROC of 0.845, which exceeded the performance of conventional urine cytology (26). Overall, integrated detection platforms combining utDNA and exosome analysis provide a more comprehensive coverage of molecular alterations in BC. These approaches offer important opportunities to improve diagnostic accuracy, monitor treatment response, and guide personalized therapeutic strategies. Looking ahead, the integration of multi-omics datasets with advanced machine learning algorithms is expected to further enhance the clinical utility of combined utDNA and exosome assays in BC management.

## 3 Clinical applications of combined detection in the classification and staging of BC

### 3.1 Diagnostic and classification value in NMIBC

NMIBC is characterized by a high recurrence rate and a substantial risk of progression to muscle-invasive disease. Therefore, early detection and accurate molecular characterization are essential for optimal clinical management. Conventional diagnostic approaches, including cystoscopy and urine cytology, remain widely used but have several limitations. Cystoscopy is invasive and associated with poor patient compliance during repeated surveillance, whereas urine cytology shows limited sensitivity, particularly for low-grade tumors. In recent years, urinary liquid biopsy approaches based on utDNA and exosomes have emerged as promising non-invasive diagnostic strategies for NMIBC. These assays provide molecular information directly derived from tumor cells and may complement conventional diagnostic methods. Multiple studies have demonstrated that utDNA reflects the genomic landscape of bladder tumors. Hirotsu et al. performed targeted sequencing of 71 genes using urine and plasma samples from 25 BC patients. Tumor-associated mutations were detected much more frequently in urine than in plasma (53% vs. 2%).

Moreover, the variant allele frequency of utDNA was significantly associated with tumor invasiveness and urine cytology results, indicating high diagnostic sensitivity for early-stage NMIBC (37). Similarly, Vedeld et al. conducted a prospective study using the BladMetrix methylation panel to detect recurrence in NMIBC patients. The assay achieved an overall sensitivity level of 91% and reached 100% sensitivity for T1, T2, and carcinoma *in situ* (CIS). Importantly, utDNA-based testing detected recurrence earlier than cystoscopy in several cases, suggesting that this approach may reduce the need for frequent invasive examinations (38). Urinary exosomes also represent an important source of tumor-derived molecular information. Zhou et al. reported that whole-exome sequencing of exoDNA identified driver gene mutations highly concordant with tumor tissue DNA. Interestingly, several mutations were detectable only in exoDNA, highlighting its ability to capture tumor heterogeneity and MRD (27). Proteomic analyses further support the clinical relevance of urinary exosomes. Hiltbrunner et al. showed that urinary exosomes retained oncogenic metabolic signatures even in patients who had achieved complete pathological remission.

These findings suggest that exosome-derived biomarkers may provide early signals of tumor recurrence or metastatic potential (30). Beyond diagnosis, urinary molecular biomarkers may also facilitate the molecular subtyping of NMIBC. Tan et al. analyzed gene expression profiles from 2,411 BC samples and identified six molecular subtypes. Notably, a subset of NMIBC tumors exhibited molecular characteristics similar to MIBC, indicating that molecular classification could help identify high-risk patients requiring closer monitoring or early intervention (5). Genomic alterations frequently observed in NMIBC—including mutations in FGFR3, TERT, and ERBB2—can be detected in urinary DNA and used for risk stratification and disease classification (39). Integrating these molecular markers may improve patient selection for personalized treatment and surveillance strategies.

In addition to DNA-based markers, RNA biomarkers in urine have demonstrated promising diagnostic performance. Abazari et al. reported that mRNAs such as ERBB2, CCND1, MKI67, and MAGEA6 were significantly upregulated in urine samples from NMIBC patients. Combining these markers increased the sensitivity of urine cytology from 37% to 87% (40). Similarly, Ingelmo-Torres et al. found that the urinary expression levels of miR-140-5p and miR-92a-3p were associated with tumor progression in NMIBC, indicating their potential as prognostic biomarkers (41). Multi-marker strategies have also been explored. Todenhöfer et al. demonstrated that combining multiple urinary biomarkers—including cytology, FISH, immunocytology, and NMP22—significantly improved the recurrence detection in NMIBC, achieving negative predictive values exceeding 98% for high-risk recurrences (42). Overall, the combined analysis of utDNA and exosome-derived biomarkers provides a comprehensive representation of tumor genomic, epigenetic, and transcriptomic features. This integrated approach shows strong potential to improve NMIBC diagnosis, molecular classification, and risk stratification. With further technological optimization and large-scale clinical validation, urinary multi-omics assays may become an important non-invasive tool for NMIBC management.

### 3.2 Application in staging and classification of MIBC

MIBC is associated with aggressive biological behavior and poor clinical outcomes. Accurate staging and molecular characterization are therefore critical for treatment planning and prognostic evaluation. Conventional staging relies on imaging and histopathological examination. However, these approaches are limited in their ability to dynamically monitor disease progression and treatment response. Emerging liquid biopsy approaches based on urinary cfDNA and utDNA provide new opportunities for non-invasive staging assessment and therapeutic monitoring. Chauhan et al. performed a multi-omics analysis of urinary cfDNA using ultra-low-pass whole-genome sequencing combined with deep targeted sequencing in 74 patients with localized MIBC. This approach enabled the sensitive detection of MRD. The predictive model achieved 87% sensitivity in identifying pathological complete response.

Importantly, MRD-positive patients showed significantly worse progression-free survival and overall survival, with hazard ratios of 3.00 and 4.81, respectively (*p* < 0.01) (32). Similarly, Zhang et al. monitored the utDNA dynamics in 20 MIBC patients receiving the PD-1 inhibitor toripalimab as neoadjuvant therapy in a phase I clinical trial. Changes in utDNA levels predicted a pathological response more accurately than imaging or conventional biomarkers and helped to identify patients who might be suitable for bladder-preserving strategies (10). ctDNA has also demonstrated value in postoperative surveillance. Birkenkamp-Demtröder et al. analyzed 370 liquid biopsy samples using personalized digital droplet PCR and observed markedly elevated ctDNA levels in patients with residual disease.

Notably, ctDNA detection preceded radiological evidence of recurrence by a median of 101 days, highlighting its potential for early relapse monitoring (22). Further evidence was provided by Eraky et al., who suggested that patients with negative postoperative ctDNA results may require less intensive imaging surveillance. Such risk-adapted monitoring strategies could reduce healthcare costs and patient burden (43). Urinary exosome analysis has also been explored for the molecular classification of MIBC. Schiffer et al. developed a urinary-proteomics-based multi-peptide biomarker panel for predicting MIBC. Prospective validation showed a sensitivity level of 81% and a specificity level of 57%. The diagnostic performance improved further when combined with tumor grading (44).

In addition, Shi et al. reviewed the roles of exosomes and their molecular cargo in urine and plasma, highlighting their potential applications in molecular subtyping and treatment response monitoring for MIBC (45). Exosomes may also actively influence tumor progression. Eldh et al. identified the enrichment of inflammation-related and cancer-associated pathways in exosomal proteomes derived from bladder tissue, suggesting that exosomes could contribute to tumor recurrence and metastasis even in patients achieving pathological remission (29). Walker et al. further emphasized that urinary exosomes show promising diagnostic specificity and sensitivity for MIBC and may eventually reduce the reliance on certain invasive diagnostic procedures (46). Despite these promising findings, current evidence supporting urinary biomarkers for the molecular classification of MIBC remains largely exploratory. Many studies involve relatively small sample sizes and lack independent external validation cohorts. In addition, different studies employ heterogeneous molecular classification frameworks, which limits comparability and reproducibility. Therefore, standardized classification systems and large prospective validation studies are still required.

### 3.3 Complementary role with histology and imaging

Although urinary molecular biomarkers have shown considerable promise, histopathological examination remains the gold standard for the diagnosis and staging of BC. Tissue biopsy provides direct morphological evaluation but may be affected by sampling bias and intratumoral heterogeneity. Imaging techniques such as CT and MRI are widely used to assess tumor invasion and distant metastasis.

However, their sensitivity is limited in detecting small lesions, and repeated imaging exposes patients to radiation and high healthcare costs. Urinary liquid biopsy offers a complementary approach by enabling non-invasive molecular profiling. Zhang et al. performed high-throughput sequencing of tumor tissue DNA, utDNA, and plasma ctDNA in 59 BC patients. utDNA showed strong concordance with tumor tissue DNA in terms of tumor cell fraction, variant allele frequency, and CNVs. The assay achieved 86.7% sensitivity, 99.3% specificity, and 99.1% overall diagnostic accuracy, outperforming plasma ctDNA (19). Strandgaard et al. further demonstrated that utDNA levels correlate with tumor burden and intratumoral field effects. Changes in utDNA were associated with tumor progression, immune responses, and clinical outcomes, supporting its value in the dynamic monitoring of tumor biology (47). Combining imaging with liquid biopsy may improve the early detection of recurrence and metastasis. Birkenkamp-Demtröder et al. reported that ctDNA detection preceded the radiological evidence of recurrence and correlated with treatment response, indicating that molecular monitoring could complement imaging during follow-up (10). Similarly, Eraky et al. suggested that ctDNA-negative patients might undergo less frequent imaging surveillance, reducing healthcare resource utilization (43). Urinary exosome profiling provides additional molecular information that is not readily captured by conventional diagnostic methods. Proteomic analyses can reveal tumor-associated proteins and signaling pathways related to the tumor microenvironment and metabolic status (29).

Moreover, Zhou et al. identified unique driver mutations in urinary exoDNA, highlighting its ability to reflect tumor heterogeneity (27). Overall, utDNA and exosome-based assays complement histology and imaging by providing dynamic molecular insights into tumor biology. Integrating these modalities may improve diagnostic accuracy, optimize surveillance strategies, and support personalized treatment planning. Conventional cystoscopy remains the primary diagnostic tool for BC detection, with reported sensitivity levels ranging from approximately 85% to 95% for visible lesions. However, cystoscopy is invasive and may have limited sensitivity for flat CIS. Urine cytology is highly specific—often exceeding 90%—but its sensitivity for low-grade tumors is frequently below 50%. In comparison, emerging studies evaluating combined utDNA and exosome assays report sensitivity levels ranging from approximately 75% to over 90%. These assays show improved detection of early-stage and low-grade tumors compared with cytology alone.

However, most available data are derived from retrospective or exploratory studies rather than large prospective head-to-head trials against cystoscopy. Therefore, although combined urinary biomarker approaches show promising diagnostic performance, they should currently be regarded as complementary rather than replacement strategies for conventional diagnostic methods. Methodological limitations must also be considered. Many studies involve relatively small cohorts and are conducted in single institutions. Patient selection bias may occur because high-risk or previously diagnosed BC populations are often overrepresented. In addition, reference standards differ across studies, with some using cystoscopy alone and others relying on histopathological confirmation. These factors may influence the reported sensitivity and specificity. Consequently, large prospective multicenter studies are required before routine clinical implementation can be recommended.

### 3.4 FDA-approved urinary biomarkers: current status and limitations

Several urinary biomarkers have received regulatory approval and are currently used in clinical practice, including NMP22, UroVysion, BTA stat, and Cxbladder. These assays generally demonstrate higher sensitivity than urine cytology for certain tumor subtypes, particularly high-grade diseases.

However, improved sensitivity is often accompanied by reduced specificity. False-positive results may occur in the presence of inflammation, hematuria, or recent urinary tract instrumentation. As a result, most approved urinary tests are used as adjunctive tools rather than standalone diagnostic methods and have not replaced cystoscopy in routine clinical practice. Compared with these established assays, the combined detection of utDNA and exosomes offers several theoretical advantages.

These approaches can simultaneously capture tumor genomic alterations and extracellular-vesicle-derived molecular signatures, potentially providing a more comprehensive representation of tumor biology. Nevertheless, unlike FDA-approved urinary biomarkers, combined utDNA and exosome assays currently lack standardized detection platforms, regulatory approval, and large-scale prospective clinical validation. Further multicenter studies are therefore necessary to establish their clinical utility and facilitate regulatory translation.

## 4 Value of combined detection of utDNA and exosomes in the clinical management of BC

### 4.1 Early screening and diagnosis

Early detection of BC is essential to improve clinical outcomes and enable timely therapeutic intervention. Conventional diagnostic approaches, including urine cytology and cystoscopy, remain the cornerstone of BC detection. However, cystoscopy is invasive and costly, whereas urine cytology demonstrates limited sensitivity, particularly for low-grade tumors. These limitations highlight the need for reliable non-invasive diagnostic tools. Recent advances in urinary liquid biopsy have identified utDNA and urinary exosomes as promising biomarkers for early BC detection. These biomarkers provide tumor-derived molecular information and can be detected through non-invasive urine sampling. Several studies have demonstrated the high diagnostic performance of utDNA-based assays. Chen et al. developed the utMeMA method, which utilizes multi-region DNA methylation analysis for early BC detection. The assay achieved a sensitivity level of 90.0% and a specificity level of 83.1%, outperforming conventional urine cytology and FISH, particularly in detecting early-stage tumors (Ta and low-grade lesions) and MRD (21).

Similarly, Yang et al. established the utLIFE-UC bioinformatic model based on utDNA alterations. This model achieved a sensitivity level of 92.8% and a specificity level of 96.0%, exceeding the diagnostic performance of cytology and FISH in identifying early UC and minimal residual lesions (33). Genomic profiling studies further support the value of utDNA detection. Hirotsu et al. performed targeted sequencing of 71 genes in urine samples and reported substantially higher detection sensitivity compared with plasma-based assays. The detected mutations closely reflected tumor mutational profiles, particularly in NMIBC patients (37).

In addition to utDNA, urinary exosomes have been widely investigated as diagnostic biomarkers. Exosomes contain diverse molecular components, including proteins and RNA molecules that originate from tumor cells. Liu et al. summarized evidence showing that molecular markers within urinary exosomes can reflect tumor development and progression, highlighting their potential for early BC detection (24). Murakami et al. identified a significantly increased expression of SLC2A1, GPRC5A, and KRT17 mRNA in urinary exosomes from BC patients, suggesting their potential as diagnostic biomarkers (28).

Similarly, Hiltbrunner et al. demonstrated that exosomes with oncogenic phenotypes could still be detected in urine even after pathological complete response, indicating their potential role in early recurrence detection (30). Combining multiple molecular markers may further improve diagnostic performance. Ou et al. analyzed mutations in five to seven genes from urinary supernatant and pellet fractions and constructed diagnostic models with AUC values of 0.94 and 0.91, respectively (48). Chung et al. applied proteomic analysis to identify differentially expressed urinary proteins and developed a diagnostic panel including APOL1 and ITIH3, achieving AUC values of 0.96 in the training cohort and 0.92 in the validation cohort (49). Multi-omics analysis has also demonstrated promising results. Chauhan et al. performed an integrated analysis of urinary cfDNA and developed a predictive model capable of detecting MRD and estimating patient survival outcomes (32). Evidence from prospective clinical studies further supports the clinical utility of urinary DNA testing. Dahmcke et al. reported a prospective study in patients presenting with gross hematuria, demonstrating a sensitivity level of 97.0% and a specificity level of 76.9% for urinary DNA testing. This approach effectively identified BC cases and reduced unnecessary cystoscopic examinations (50). Rose et al. also highlighted that utDNA assays, as a form of liquid biopsy, may overcome several limitations of tissue biopsy and facilitate the precision management of BC (51). Collectively, these studies suggest that utDNA and exosome-based biomarkers offer promising non-invasive strategies for early BC detection. Compared with traditional diagnostic methods, these molecular assays demonstrate improved sensitivity and may reduce the need for invasive procedures. Representative studies evaluating their diagnostic performance are summarized in **Table 1**.

**Table 1:** Comparison of conventional diagnostic methods and emerging liquid biopsy approaches for BC.

Diagnostic method	Biomarker/principle	Sensitivity	Specificity	Invasiveness	Cost	Clinical application	Key limitations
Cystoscopy	Endoscopic visualization of bladder mucosa	85%–95%	85%–90%	Invasive	High	Gold standard for diagnosis and surveillance	Patient discomfort, high cost, limited sensitivity for flat lesions
Urine cytology	Morphological evaluation of exfoliated tumor cells	~42% overall (high for high-grade tumors)	95%–100%	Non-invasive	Low	Detection of high-grade tumors and CIS	Very low sensitivity for low-grade tumors
NMP22	Nuclear matrix protein released from tumor cells	47%–85%	60%–90%	Non-invasive	Moderate	Adjunctive test for diagnosis and surveillance	False positives in infection or hematuria
UroVysion FISH	Chromosomal aberration detection (3, 7, 17; 9p21 loss)	63%–83%	85%–87%	Non-invasive	High	Detection of recurrence and equivocal cytology	Requires specialized laboratory infrastructure
RNA-based urine tests (e.g., Cxbladder)	mRNA expression panels	80%–85%	80%–90%	Non-invasive	Moderate–high	Hematuria evaluation and NMIBC surveillance	Cost and limited guideline integration
utDNA analysis	Tumor-derived DNA mutations in urine	80%–90%	85%–96%	Non-invasive	Moderate	Molecular detection and early diagnosis	Lack of large prospective validation
Urinary exosome biomarkers	Exosomal RNA/protein signatures	~75%	~77%	Non-invasive	Moderate	Tumor heterogeneity assessment and biomarker discovery	Lack of assay standardization
Combined utDNA + exosome assays	Integrated genomic and extracellular vesicle biomarkers	90%–95% (reported in exploratory studies)	90–96%	Non-invasive	Moderate–high	Emerging liquid biopsy for diagnosis, surveillance, and MRD detection	Limited clinical validation

### 4.2 Efficacy monitoring and recurrence prediction

BC is characterized by a high recurrence rate after treatment, particularly among patients with NMIBC. Approximately half of NMIBC patients experience tumor recurrence, and a subset may progress to MIBC. Therefore, reliable biomarkers to monitor therapeutic response and predict recurrence are essential to optimize patient management. utDNA and exosome-based assays have shown considerable potential for the dynamic monitoring of treatment efficacy and recurrence risk. Birkenkamp-Demtröder et al. applied personalized digital droplet PCR to detect tumor DNA in urine and plasma samples. Patients with disease recurrence exhibited significantly higher utDNA levels compared with disease-free individuals.

Importantly, increases in tumor DNA were observed before the clinical evidence of progression, suggesting that utDNA may serve as an early biomarker for recurrence detection (12). Further work from the same research group demonstrated that ctDNA detection predicted metastatic recurrence a median of 101 days before radiological diagnosis following radical cystectomy. ctDNA levels were also closely associated with treatment response (22). Liquid biopsy approaches may also help guide postoperative treatment decisions. Powles et al. reported that ctDNA assays can identify patients with molecular residual disease after surgery, allowing clinicians to select individuals who may benefit from adjuvant immunotherapy (52).

Similarly, Chauhan et al. constructed a random forest model based on the multi-omics analysis of urinary cfDNA to predict the MRD status. The model achieved 87% sensitivity, and MRD-positive patients exhibited significantly worse progression-free and overall survival, demonstrating the prognostic value of urinary DNA analysis (32). Dynamic monitoring of utDNA may also be useful to evaluate immunotherapy response. Zhang et al. assessed utDNA dynamics in MIBC patients receiving neoadjuvant immunotherapy with toripalimab. Changes in utDNA levels predicted pathological complete response more accurately than imaging or conventional biomarkers (10). Urinary-exosome-derived biomarkers have also shown potential in predicting disease progression. Ingelmo-Torres et al. identified miR-140-5p and miR-92a-3p as independent predictors of tumor progression in NMIBC (41). Hiltbrunner et al. further reported that oncogenic exosomes could persist in urine even after apparent tumor remission, suggesting their possible role in recurrence monitoring (30). Technological advances have further improved detection performance. Lee et al. developed the uAL100 method based on targeted deep sequencing of urinary DNA. This assay achieved 83.7% sensitivity and 100% specificity for BC detection and reduced unnecessary cystoscopies (36).

In addition, multi-marker strategies may improve surveillance accuracy. Todenhöfer et al. demonstrated that combining urinary biomarkers—including cytology, FISH, and immunocytology—significantly enhanced the detection of high-risk recurrence, achieving negative predictive values exceeding 99% (42). Overall, these findings indicate that the combined analysis of utDNA and exosome-derived biomarkers provides a powerful tool to monitor therapeutic response and predict recurrence risk in BC patients.

### 4.3 Postoperative follow-up and MRD detection

Postoperative surveillance aims to detect tumor recurrence and MRD at the earliest possible stage. Conventional follow-up strategies rely heavily on cystoscopy and imaging. However, these methods are invasive, costly, and limited in their ability to detect early molecular changes. Urinary liquid biopsy offers a non-invasive alternative for postoperative monitoring. Vedeld et al. conducted a prospective study using the BladMetrix DNA methylation biomarker panel to detect utDNA in urine samples. The assay achieved a sensitivity level of 91% for recurrence detection, including all T1, T2, and CIS cases, with a false-negative rate below 1%.

Importantly, this approach could potentially reduce cystoscopy procedures by approximately 55%, thereby improving patient compliance and optimizing follow-up schedules (38). Similarly, Eraky et al. applied the tumor-informed ctDNA assay Signatera™ and found that patients who remained ctDNA-negative after surgery had a significantly lower risk of recurrence. These findings suggest that follow-up imaging intensity may be safely reduced in low-risk patients (43). Chauhan et al. also demonstrated that preoperative utDNA levels were significantly associated with postoperative pathological complete response. MRD-positive patients exhibited a markedly worse progression-free survival, highlighting the role of utDNA in postoperative risk stratification (11). Zhang et al. further showed that dynamic monitoring of utDNA could guide bladder-preserving treatment decisions following neoadjuvant immunotherapy, suggesting a potential role for urinary DNA testing in postoperative therapeutic management (10). Urinary exosome research also supports its application in postoperative surveillance. Hiltbrunner et al. observed that persistent oncogenic exosomes in urine may indicate MRD, suggesting their potential as biomarkers for recurrence risk assessment (30).

Additionally, urinary miRNA and proteomic analyses have identified several molecular candidates for postoperative recurrence monitoring, further expanding the range of available biomarkers (41). Overall, combined utDNA and exosome assays provide a sensitive and non-invasive approach for postoperative follow-up and MRD detection in BC patients. These strategies may help optimize surveillance schedules, reduce unnecessary invasive procedures, and support individualized patient management.

However, several limitations should be considered. Most studies evaluating urinary biomarkers for recurrence monitoring are retrospective and observational in design. Sample sizes are often limited, and follow-up durations may be relatively short. In addition, variations in treatment regimens and recurrence definitions across studies introduce methodological heterogeneity. Therefore, well-designed prospective multicenter trials with standardized endpoints are required to establish the clinical utility and cost-effectiveness of these biomarkers. Representative studies evaluating utDNA and exosome-based biomarkers across different clinical contexts are summarized in **Table 2**.

**Table 2:** Representative studies evaluating utDNA and exosomes in bladder cancer across different clinical applications (NMIBC, MIBC, and MRD).

Clinical application	Study	Biomarker type	Sample size	Key findings	Sensitivity	Specificity	Limitations
NMIBC detection	Dudley et al., 2019 (31)	utDNA mutation panel	76	utDNA mutations detected bladder cancer with higher sensitivity than cytology	83%	96%	Small cohort; retrospective
	Springer et al., 2018 (53)	UroSEEK assay	570	Multi-gene utDNA panel improved early detection of NMIBC	83%	93%	Limited prospective validation
	Ward et al., 2016 (54)	Exosomal miRNA panel	85	Urinary exosomal miRNAs discriminated NMIBC from controls	81%	85%	Heterogeneous patient population
MIBC diagnosis	Christensen et al., 2019 (55)	utDNA sequencing	102	utDNA detected genomic alterations associated with MIBC	89%	94%	Retrospective design
	Hiltbrunner et al., 2020 (30)	Exosomal RNA signature	60	Exosome-derived RNA correlated with tumor stage and grade	78%	88%	Small sample size
MRD	Birkenkamp-Demtröder et al., 2017 (22)	utDNA mutation tracking	68	utDNA detected recurrence months before clinical diagnosis	92%	90%	Lack of external validation
	Chauhan et al., 2021 (11)	Combined utDNA + exosomal biomarkers	54	Combined biomarkers improved MRD detection accuracy	94%	91%	Exploratory study

## 5 Standardization procedures and current status of clinical translation

### 5.1 Standardization of sample collection, processing, and detection procedures

The combined detection of utDNA and exosomes has demonstrated promising value in the molecular subtyping, staging, and clinical management of BC. However, the successful clinical implementation of these assays depends heavily on the standardization of sample collection, processing, and detection workflows.

Urine represents an attractive liquid biopsy sample because of its non-invasive nature and the possibility of repeated self-collection. Nevertheless, the concentration and stability of tumor-derived nucleic acids in urine are highly susceptible to pre-analytical variables, including collection methods, storage conditions, and transport procedures. Wever et al. emphasized that these factors can significantly influence the integrity of urinary DNA and RNA, thereby affecting downstream molecular analyses (56). To address these challenges, standardized protocols for urine handling have been proposed. These protocols recommend minimizing contamination during collection, using dedicated preservation solutions, and maintaining controlled transport temperatures to preserve nucleic acid integrity. Such measures are essential to ensure reliable and reproducible detection results.

Pre-analytical processing steps also play a critical role in assay performance. Ou et al. highlighted the distinct biological characteristics of cfDNA in the urine supernatant and cellular DNA in the urinary sediment. Both fractions may contain tumor-derived genetic material but require different processing strategies. Using high-sensitivity NGS combined with multi-gene panels—including TERT, FGFR3, and TP53—the authors achieved high diagnostic accuracy for BC (48). Their study further emphasized the importance of standardized centrifugation parameters, including speed and duration, as well as appropriate preservation conditions. These factors are essential to minimize nucleic acid degradation and prevent contamination, thereby ensuring high assay sensitivity and specificity.

Technological advances are also contributing to the improvement of standardized workflows. Chen et al. reviewed recent developments in urinary liquid biopsy technologies and highlighted the growing role of microfluidic platforms and artificial-intelligence-assisted data analysis in improving sample processing efficiency and analytical accuracy (35). They suggested that future standardization efforts should cover the entire workflow, from sample collection and preservation to nucleic acid extraction and molecular detection. In addition, Kretschmer-Kazemi Far et al. proposed a standardized RT-qPCR-based workflow for urinary RNA detection. Their protocol provides detailed procedures for sample collection, stabilization, transport, and RNA extraction, enabling the reliable and reproducible detection of urinary RNA biomarkers in BC patients (57). Taken together, current standardization efforts for utDNA and exosome detection generally involve several key elements as follows: (1) minimizing contamination during urine collection and using dedicated preservation solutions, (2) maintaining controlled transport temperatures to prevent nucleic acid degradation, (3) standardizing centrifugation and separation procedures to preserve cfDNA and exosome integrity, (4) applying high-sensitivity detection technologies such as NGS and digital PCR, and (5) establishing uniform quality control standards to ensure assay reproducibility and accuracy. The implementation of standardized workflows is a fundamental prerequisite for the reliable clinical application of urinary liquid biopsy assays in BC management.

### 5.2 Data interpretation, quality control, and multicenter validation

Beyond technical standardization, the clinical translation of utDNA and exosome assays also requires reliable data interpretation frameworks, robust quality control systems, and validation in large multicenter cohorts. Dang and Park highlighted that although ctDNA and related liquid biopsy technologies offer important advantages for non-invasive disease monitoring, several analytical challenges remain. These include complex variant interpretation, the potential for false-positive and false-negative results, and variability in detection sensitivity across platforms. The authors emphasized the need for standardized bioinformatics pipelines and quality control procedures to ensure the reliability of clinical test results (58).

Several studies have proposed analytical frameworks to address these challenges. Yang et al. developed the utLIFE-UC model, which integrates BC-specific mutations with large-scale CNVs to enhance utDNA detection. The model achieved a sensitivity level of 92.8% and a specificity level of 96.0% in clinical cohorts. Validation using datasets from The Cancer Genome Atlas (TCGA) and upper tract UC further demonstrated its strong generalizability (33). Similarly, Lee et al. introduced the uAL100 approach, which uses targeted deep sequencing to identify tumor-specific mutations in urinary DNA. The assay achieved 83.7% sensitivity and 100% specificity. By incorporating stringent bioinformatic filtering strategies, the method effectively reduced the background noise and minimized the false-positive variant calls (36).

These findings highlight the importance of standardized variant calling algorithms and background noise control strategies in urinary DNA analysis. In addition to analytical standardization, multicenter validation is essential for clinical translation. Kravchuk et al. performed a systematic evaluation of urinary DNA testing and reported that the Uromonitor system achieved 80.2% sensitivity and 96.9% specificity to detect NMIBC recurrence. Aggregated multicenter data confirmed the stability and reproducibility of the assay across different clinical settings (34). Quality control procedures should therefore encompass the entire analytical workflow, including sample collection, nucleic acid extraction, sequencing, and downstream data analysis. Green et al. further emphasized that multicenter clinical trials are critical to validate the diagnostic and prognostic value of liquid biopsy technologies in BC. Standardized quality control frameworks and unified data interpretation guidelines are necessary prerequisites for their integration into clinical practice (59).

Overall, the successful clinical adoption of utDNA and exosome assays requires comprehensive frameworks for variant detection, standardized bioinformatics pipelines, and robust quality control systems. Large multicenter validation studies will be essential to confirm their reproducibility and clinical utility.

### 5.3 Review of existing guidelines and standardized pathways

The current clinical guidelines for BC diagnosis and management have begun to acknowledge the potential of urinary molecular biomarkers, including utDNA and exosome-based assays.

However, their routine clinical implementation remains limited due to the need for stronger clinical evidence. The European Association of Urology guidelines recommend the TNM staging system and the WHO grading system as the standard framework for BC diagnosis and management. Although urinary biomarkers are discussed within these guidelines, their clinical use remains cautious, reflecting the current lack of high-level evidence for routine implementation (9). Liedberg et al., representing the EAU NMIBC guideline panel, emphasized that ongoing non-inferiority randomized controlled trials are evaluating the safety of urine-biomarker-guided surveillance strategies. These trials aim to determine whether biomarker-based monitoring could safely reduce the frequency of cystoscopy during follow-up (60). The authors also highlighted that urinary biomarkers must comply with European *in vitro* diagnostic regulatory requirements and demonstrate clear cost-effectiveness before being incorporated into routine clinical recommendations. Burchardt et al. reviewed the development of BC biomarker technologies and concluded that although urinary biomarkers demonstrate promising sensitivity and specificity, no single biomarker currently has sufficient evidence to replace cystoscopy or urine cytology. The current guidelines recommend using urinary biomarkers instead as adjunctive tools in combination with clinical and pathological information (61).

Similarly, Svatek et al. emphasized that BC screening strategies based on urinary biomarkers must be supported by strong clinical evidence and cost-effectiveness analyses to avoid overdiagnosis and unnecessary healthcare burden. Large multicenter validation studies are therefore required before widespread screening implementation can be considered (62). Ecke et al. further noted that advances in molecular diagnostics may enable more precise molecular subtyping and personalized treatment strategies in the future. Nevertheless, standardized detection platforms and robust clinical validation remain essential prerequisites for clinical adoption (63).

Overall, current clinical guidelines adopt a cautious but encouraging stance toward urinary molecular biomarkers. The integration of utDNA and exosome detection into routine clinical practice will require standardized analytical workflows, compliance with regulatory frameworks, and strong evidence from large prospective clinical trials. To further enhance the transparency and completeness of this review, a supplementary file entitled “Summary of Ongoing and Completed Clinical Trials Involving Urinary Tumor DNA and Exosome Detection in BC” has been provided. This file summarizes key clinical trials retrieved from ClinicalTrials.gov and the Chinese Clinical Trial Registry, including study design, sample size, biomarker type, detection platform, and primary outcomes. These data provide an updated overview of the current translational progress of urinary liquid biopsy technologies in BC management.

## 6 Challenges, innovations, and future development trends

### 6.1 Key challenges and technical bottlenecks currently faced

The combined detection of utDNA and urinary exosomes represents a promising non-invasive approach for BC diagnosis and monitoring. However, several technical and translational challenges still limit its widespread clinical adoption. One major limitation is the relatively low abundance of tumor-derived DNA in urine. This is particularly evident in patients with early-stage disease or low tumor burden, where detection sensitivity may be insufficient. Smith et al. reported that the ctDNA levels in plasma and urine of patients with renal tumors were generally low, with urinary ctDNA detected in only approximately 50% of cases. Detection rates were associated with tumor size and the presence of vascular tumor thrombi, indicating that improved assay sensitivity is required for reliable clinical application (20).

Another challenge arises from the complex composition of urine. Urine contains substantial amounts of non-tumor-derived DNA and other biomolecules, which may interfere with molecular detection and increase the risk of false-positive results. Wever and Steenbergen highlighted that biological factors such as urinary pH and enzymatic activity may influence DNA stability, complicating the extraction and purification of tumor-derived cfDNA (56). These factors underscore the need for optimized sample processing and preservation protocols. Technical standardization remains another critical challenge. Although high-throughput sequencing and digital PCR technologies have significantly improved detection sensitivity, variability among laboratories in terms of analytical platforms, bioinformatic pipelines, and threshold settings can compromise reproducibility. Dang and Park emphasized that such differences may affect data interpretation and clinical decision-making, highlighting the need for standardized analytical frameworks (58).

In addition, the current exosome isolation techniques are not yet fully optimized. Methods such as ultracentrifugation, polymer precipitation, and immunoaffinity capture each have advantages and limitations in terms of efficiency, purity, and scalability. Yin et al. noted that the lack of standardized protocols for exosome isolation remains a major barrier to widespread clinical application (23). Biological heterogeneity further complicates biomarker development. BC comprises multiple molecular subtypes and diverse pathological features, making it unlikely that a single biomarker can capture the full spectrum of tumor characteristics. Maas et al. emphasized that integrated multi-omics approaches are therefore required, although such strategies introduce additional challenges in data integration and interpretation (64).

Moreover, utDNA fragments are typically highly degraded and may be influenced by non-tumor factors such as urinary tract infections or inflammatory conditions. These factors may affect detection specificity and analytical accuracy (65). Finally, several practical obstacles limit clinical translation. These include relatively high assay costs, long turnaround times, and limited large-scale clinical validation. Carr and Welch noted that although ctDNA-based assays show considerable promise, randomized controlled trials evaluating their impact on patient outcomes remain limited. The complexity and cost of the current workflows also hinder routine clinical implementation (66). Overall, addressing these technical, biological, and logistical challenges will be essential for the successful integration of utDNA and exosome-based assays into clinical practice.

### 6.2 Emerging integrated platforms and personalized medicine prospects

To overcome these limitations, several innovative analytical platforms have been developed in recent years, improving the sensitivity and clinical applicability of urinary liquid biopsy technologies. Yang et al. developed the utLIFE-UC model, which integrates tumor-specific mutations with large-scale CNVs detected in urine. This model achieved a sensitivity level of 92.8% and a specificity level of 96.0% in detecting UC. Importantly, it outperformed conventional urine cytology and FISH in monitoring MRD (33).

Similarly, Chauhan et al. applied a multi-omics strategy that combined ultra-low-pass whole-genome sequencing with deep targeted sequencing of urinary cfDNA. This approach enabled sensitive MRD detection in patients with localized BC and accurately predicted both progression-free survival and overall survival (32). Advances in computational methods are also transforming urinary biomarker analysis. Chen et al. highlighted the potential of microfluidic technologies and artificial intelligence algorithms for automated sample processing and high-dimensional data interpretation, thereby facilitating precision diagnostics (35). Gerke et al. further proposed that multi-parameter models integrating ctDNA profiles with clinical and molecular data may enable dynamic patient monitoring and individualized therapeutic decision-making (67). In the exosome field, Yin et al. emphasized that exosomes contain a diverse array of biomolecules—including proteins, mRNA, and miRNA—that reflect tumor biology.

In addition to their diagnostic potential, exosomes may also serve as delivery vehicles for targeted therapies, offering new opportunities for precision medicine (23). Kravchuk et al. reported promising results from the Uromonitor system, which detects urinary DNA mutations and demonstrated a sensitivity level of 80.2% and a specificity level of 96.9% in NMIBC patients. These results significantly exceeded those of urine cytology and support the role of urinary DNA testing as an auxiliary diagnostic tool (34). Another emerging trend is the integration of multiple biomarker classes. Maas et al. suggested that combining urinary genomic, proteomic, and epigenetic markers may overcome the limitations of single-biomarker approaches and improve diagnostic and prognostic accuracy (64).

Similarly, Shi et al. highlighted that exosome-based multi-omics analyses can provide a more comprehensive view of tumor heterogeneity and support personalized treatment strategies (45). Taken together, these technological advances are gradually improving the diagnostic performance and clinical applicability of urinary liquid biopsy platforms.

### 6.3 Implementation challenges and health system considerations

Despite promising diagnostic performance, several practical barriers must be addressed before combined utDNA and exosome assays can be integrated into routine clinical practice. Cost represents a major limitation, particularly for NGS-based platforms required for high-sensitivity mutation detection. Compared with urine cytology, which is relatively inexpensive, molecular assays may significantly increase the per-patient healthcare expenditure.

Turnaround time and laboratory infrastructure requirements represent additional challenges. High-throughput sequencing, exosome isolation, and bioinformatic analysis require specialized equipment and trained personnel, which may not be available in all clinical settings, especially in resource-limited regions. Regulatory approval pathways also remain unclear for combined biomarker platforms.

Although several urinary biomarkers have received regulatory clearance, integrated utDNA–exosome assays currently lack standardized protocols and large-scale multicenter validation necessary for regulatory approval. Finally, reimbursement policies remain uncertain. Without clear evidence demonstrating reduced cystoscopy frequency, improved survival outcomes, or overall healthcare cost savings, widespread reimbursement is unlikely. Prospective health economic analyses will therefore be critical for real-world clinical adoption.

### 6.4 Potential near-term clinical applications

Although complete replacement of cystoscopy is unlikely in the near future, several clinical scenarios may represent feasible early adoption settings for urinary liquid biopsy technologies. One promising application is the surveillance of high-risk NMIBC patients. These individuals require frequent cystoscopic examinations because of high recurrence rates. In this context, urinary biomarker assays could serve as complementary tools to reduce cystoscopy frequency or identify patients requiring intensified monitoring.

Another important application is the detection of MRD after radical cystectomy or bladder-preserving therapy. Early identification of molecular relapse before radiological recurrence may allow earlier therapeutic intervention and improved risk stratification.

Additional applications may include perioperative risk assessment and monitoring of treatment response during intravesical therapy. These targeted clinical scenarios may facilitate gradual clinical integration while larger validation studies are conducted.

### 6.5 Future research directions and prospects for clinical translation

Future research should focus on improving the analytical sensitivity and specificity of combined utDNA and exosome detection while optimizing sample processing and analytical workflows. Chauhan et al. demonstrated the feasibility of non-invasive MRD detection in MIBC patients using uCAPP-Seq, suggesting that increased sequencing depth and broader genomic coverage may further enhance detection performance (11). Large-scale prospective clinical trials will be essential to validate the clinical utility of urinary biomarkers across different risk-stratified patient populations. Maas et al. emphasized that multicenter studies are critical to establish robust clinical evidence and facilitate guideline adoption (60). Future studies should also explore the integration of molecular subtyping with immunophenotypic profiling. Lokeshwar et al. highlighted that molecular subtypes of BC are closely associated with treatment response, and urinary liquid biopsy could support the dynamic monitoring of therapeutic efficacy (6).

Similarly, Heard et al. emphasized the importance of developing biomarker panels with stronger predictive value to guide treatment selection and evaluate responses to emerging therapies (68). From a translational perspective, Garofoli et al. proposed that integrating ctDNA-based assays into clinical practice requires multidisciplinary collaboration among clinicians, molecular biologists, and bioinformaticians to ensure accurate interpretation and appropriate clinical application (69).

Furthermore, Maas et al. noted that urinary biomarker assays meeting *in vitro* diagnostic certification requirements and demonstrating cost-effectiveness are more likely to be incorporated into clinical guidelines (60). In summary, the combined detection of utDNA and urinary exosomes is rapidly evolving as a promising strategy for non-invasive BC management. Continued technological innovation, rigorous clinical validation, and multidisciplinary collaboration will be essential to facilitate its translation into precision diagnostics and individualized therapeutic strategies.

## 7 Conclusion

Urinary liquid biopsy has emerged as a promising non-invasive strategy for the detection and monitoring of BC. Among the currently investigated approaches, utDNA and exosome-derived biomarkers represent two complementary sources of tumor-associated molecular information. utDNA enables the detection of tumor-specific genomic and epigenetic alterations, whereas exosomes carry diverse biomolecules, including nucleic acids and proteins, that may reflect tumor heterogeneity and dynamic tumor–host interactions. Accumulating evidence suggests that the combined analysis of utDNA and exosomal biomarkers may provide improved diagnostic and prognostic performance compared with single-marker assays. This integrated approach has shown potential applications in several clinically relevant scenarios, including early tumor detection, recurrence surveillance, and MRD monitoring. Such strategies may be particularly valuable in settings that currently rely on repeated invasive procedures, such as surveillance of NMIBC and post-treatment monitoring following radical cystectomy. Despite these encouraging developments, several challenges must be addressed before these technologies can be widely implemented in routine clinical practice. Many currently available studies are limited by relatively small sample sizes, retrospective study designs, and heterogeneity in analytical methodologies.

In addition, standardized protocols for urine collection, biomarker isolation, and data analysis remain insufficiently established, which may compromise reproducibility across studies. Importantly, the clinical utility of combined biomarker assays requires validation in large-scale prospective multicenter trials, ideally with direct comparisons to established diagnostic modalities such as cystoscopy, urine cytology, and regulatory-approved urinary biomarkers.

Future research should therefore prioritize the development of standardized and scalable analytical platforms, the integration of multi-omics biomarker strategies, and rigorous validation in well-characterized clinical cohorts. Furthermore, cost-effectiveness analyses and real-world implementation studies will be essential to determine how urinary liquid biopsy technologies can be effectively incorporated into routine BC management.

In summary, combined utDNA and exosome-based urinary liquid biopsy represents a promising frontier in BC diagnostics and disease monitoring. With continued technological refinement and robust clinical validation, these approaches may complement existing diagnostic tools and potentially reduce the reliance on invasive procedures, ultimately contributing to improved patient outcomes and more personalized disease management.
